# The effect of digital video feedback on roundoff learning outcomes in gymnastics learning

**DOI:** 10.3389/fspor.2026.1771596

**Published:** 2026-03-26

**Authors:** Rizal Ahmad Fauzi, Ayi Suherman, Entan Saptani, Suthana Tingsabhat, Mamat Heryanto

**Affiliations:** 1 Physical Education Study Program, Universitas Pendidikan Indonesia, Bandung, Indonesia; 2 Department of Curriculum and Instruction, Faculty of Education, Chulalongkorn University, Bangkok, Thailand; 3 Department of Physical Education, Health and Recreation STKIP Pasundan, Cimahi, West Java, Indonesia

**Keywords:** digital video, feedback, gymnastics learning, round-off, technology

## Abstract

The technological breakthroughs of the Fourth Industrial Revolution have underscored the necessity of incorporating technology into education, encompassing physical education. Nonetheless, its utilisation in gymnastics education is still restricted. This study sought to investigate the impact of digital video feedback on students' learning results in round-off gymnastics. This study utilised a quasi-experimental design using a one-group pretest-posttest methodology. A total of 36 students were randomly chosen from a sample of 141 individuals. A gymnastics skill observation checklist was employed to evaluate round-off performance. The Wilcoxon signed-rank test and effect size (r) were employed for data analysis. The findings indicated a statistically significant difference between pretest and posttest scores (*p* < 0.05). The average score rose from 1.74 to 3.67, with an impact size of r = 0.86, signifying a substantial benefit. The data indicate that digital video feedback markedly enhances students' round-off performance. Overall digital video feedback serves as an efficacious instructional instrument in gymnastics education, augmenting students' comprehension, performance, and engagement. The incorporation of technology in physical education can enhance motor skill development, especially in intricate movements like the round-off.

## 1 Introduction

Video feedback in gymnastics instruction has become a notable pedagogical instrument, especially among college students, enhancing skill acquisition and performance through diverse feedback modalities. Video feedback lets students see how they did next to an expert model, which makes for a unique learning experience that combines visual evidence with corrective information from both the student's performance and the expert's demonstration. This teaching method helps students learn complicated motor skills, like those needed for gymnastics, by helping them build their internal models and improve the techniques they use in performance (1). Somersaults, spins, and rolls are gymnastics moves that need a lot of accuracy, body awareness, and risk management. This can make people anxious and make it harder to learn new skills. Using visual feedback techniques helps students critically evaluate their performance, which helps them improve their technique and boosts their confidence (2–4).

Research indicates that knowledge imparted via video feedback is essential for refining motor skills, enabling students to visualize expert performance and utilize insights derived from observing their own actions (1). This dual approach creates an environment where learners can effectively self-assess and correct their movements, which improves their technical skills even more than those who only get verbal feedback (5, 6). Video feedback also helps teachers meet the different needs of students in larger classes, giving them chances to learn in ways that are unique to them (6, 7).

Learning skills is often necessary, as in sports training and learning or even for rehabilitation therapy (8, 9). Before they can do a new movement, each person has to go through a few steps, one of which is watching someone else do the movement. There is a distinct principle that observation can lead to the acquisition of new motor behaviors, rather than merely influencing the individual's existing motor memory (10). It necessitates phases of observation, repetition (practice), and automaticity, along with the provision of feedback, akin to acquiring gymnastics movements like round-offs.

Research has indicated that observation-based feedback effectively illustrates students’ acquisition of new motor skills and competencies (11). Several studies have demonstrated that visual information integrating expert and self-modeling typically yields superior learning outcomes, specifically enhanced skills compared to visual information derived from self-observation, self-modeling, or expert modeling alone (12–14). Incorporating video feedback into gymnastics training is a crucial element that acknowledges both the cognitive and physical dimensions of motor learning (2). In most cases, video is used to give a combination of self-observation or self-modeling and expert feedback (15–17). Numerous studies have demonstrated beneficial outcomes for self-observation videos (12, 18). Research indicated that physical education students, utilizing video-based feedback, achieved a more profound comprehension of the intended learning outcomes, simultaneously enhancing motivation and engagement in the educational process (19). This kind of visual information has been used to help people learn how to do a lot of different movements, like volleyball passes, rowing, badminton serves, or other gymnastic moves (16, 17, 20).

The steps that have been taken so far to teach round-off are not working and will take a long time to learn. This is because the teacher can't give clear feedback, which makes it hard for them to do their job as a feedback provider. This is because round-off skills are hard to learn and require you to be able to put several movements together into one. Round-off is a basic gymnastics move that helps you get faster before doing other moves like somersaults or flips (21). Teachers can't give students feedback that they can understand well enough because the concrete forms that students can see limit how complicated the movements and the material being explained can be. So, media is needed to help teachers give students the right kind of feedback. Giving students feedback while they are learning has long been shown to be an important part of helping them figure out what they should and shouldn't do (21). Some studies look at kinematic measures taken at certain points in the movement and look at very specific parts of how the movement is done, like body segment alignment, joint orientation, or paddle stroke length and rate (12, 16, 20). No studies have concentrated on delivering feedback and observation through video and motion analysis applications on smartphones. Consequently, research is required to investigate the provision of feedback in video format and the collaboration with a smartphone sports motion analysis application, as well as to evaluate the outcomes of its implementation in learning round-off movements.

## 2 Methods

### 2.1 Study design

This study utilized a quasi-experimental pretest–posttest design to investigate the impact of smartphone-based video analysis on students’ round-off motor skill performance. This design was chosen to discern variations in performance prior to and subsequent to the intervention, while preserving a regulated learning environment conducive to technology-assisted instruction (22).

### 2.2 Participants

The study involved 36 undergraduates (aged 17–19 years) participating in a physical education program at an Indonesian institution, comprising 22 males and 14 females. During the study, the students were enrolled in a gymnastics course that encompassed numerous skills not pertaining to the round-off method. Despite all participants possessing prior gymnastics experience, they had not undergone specialized training in round-off execution during the course. Every participant possessed a smartphone capable of executing video analysis software. Participation in the study was voluntary, and all consenting students completed the intervention program.

### 2.3 Intervention

The intervention took place over four weeks and included 12 teaching sessions (three sessions each week). Each session lasted about an hour and was part of regular physical education classes.

The intervention structure was set up like this:

Week 1 (Sessions 1–3): The instructor will show you how to do round-off techniques, give you a pretest, and show you how to use the video analysis app. Students watched videos of expert models and learned how to record and review their own movements.
Week 2 (Sessions 4–6): Concentrate on the round-off's approach and take-off phases. Students recorded their performances, used the video analysis app to look at how their bodies and hands were positioned, and got feedback from the teacher.
Week 3 (Sessions 7–9): Focus on the main rotation and the phase of hand support. Students used frame-by-frame and angle analysis tools to compare their movements to those of experts. It was suggested that people talk to each other and think about themselves.
Week 4 (Sessions 10–12): Putting together the whole round-off sequence, improving how you land, and making sure you always do well. During the last session, posttest evaluations were done.

In practice, each student has their own smartphone with a video analysis app (like Hudl Technique or Coach's Eye) on it, which gives them direct and personalized access to learning tools. This method is good for teaching because it lets students focus on watching, analyzing movements, and thinking about how they did on their own (23). In other words, students are in charge of their own learning, which is in line with the idea of active and personalized technology-based learning (24).

### 2.4 Instrument

The principal instrument employed in this study was a round-off skill observation checklist, crafted to evaluate students’ performance by assessing each movement phase individually. The checklist evaluates essential technical elements of the round-off, encompassing the approach phase, hand positioning, body rotation, and landing stability. Every component is paired with explicit performance standards and detailed rating rules. Students were assigned scores from 1 to 4 for each component, with a score of 4 signifying that the movement was performed accurately and consistently throughout the start, execution, and ending phases in accordance with the established criteria.

This observational checklist is frequently employed in gymnastics skill assessment to systematically evaluate motor performance based on established criteria. The instrument utilized in this study was evaluated by professionals, incorporating consultations with gymnastics and physical education authorities to guarantee content relevancy, clarity of criteria, and conformity with established technical standards for round-off performance.

The evaluations were performed by assessors experienced in gymnastics instruction, who consistently utilized the checklist for both pre-test and post-test evaluations. The checklist facilitated a systematic and objective assessment of skill performance among participants. Smartphone-based video analysis apps like Hudl Technique and Coach's Eye were used to record videos, which let users review them frame by frame and get visual feedback.

### 2.5 Statistical analysis

The data analysis was performed in multiple phases. Initially, tests for parametric assumptions, including normality and homogeneity assessments, were conducted to ascertain the suitable statistical methods. The results demonstrated that the data were not normally distributed, thus failing to satisfy the assumptions necessary for parametric testing. Thus, a non-parametric statistical method was employed. The Wilcoxon signed-rank test was employed to assess the differences between pre-test and post-test results, as illustrated in Table 2. An effect size analysis was performed using the r statistic, derived from the Z value of the Wilcoxon test, to evaluate the practical importance of the observed change.

## 3 Results

This section displays the results of data collection after the pretest and posttest. The collected data can be seen in Table 1.

**Table 1:** Description of round-Off average values.

Descriptive Statistics						
		N	Mean	Standard Deviation	Minimum	Maximum
Round Off	Pre-Test	36	1.74	.96	1.00	3.50
	Post_Test		3.67	.48	2.00	4.00

**Figure 1:**
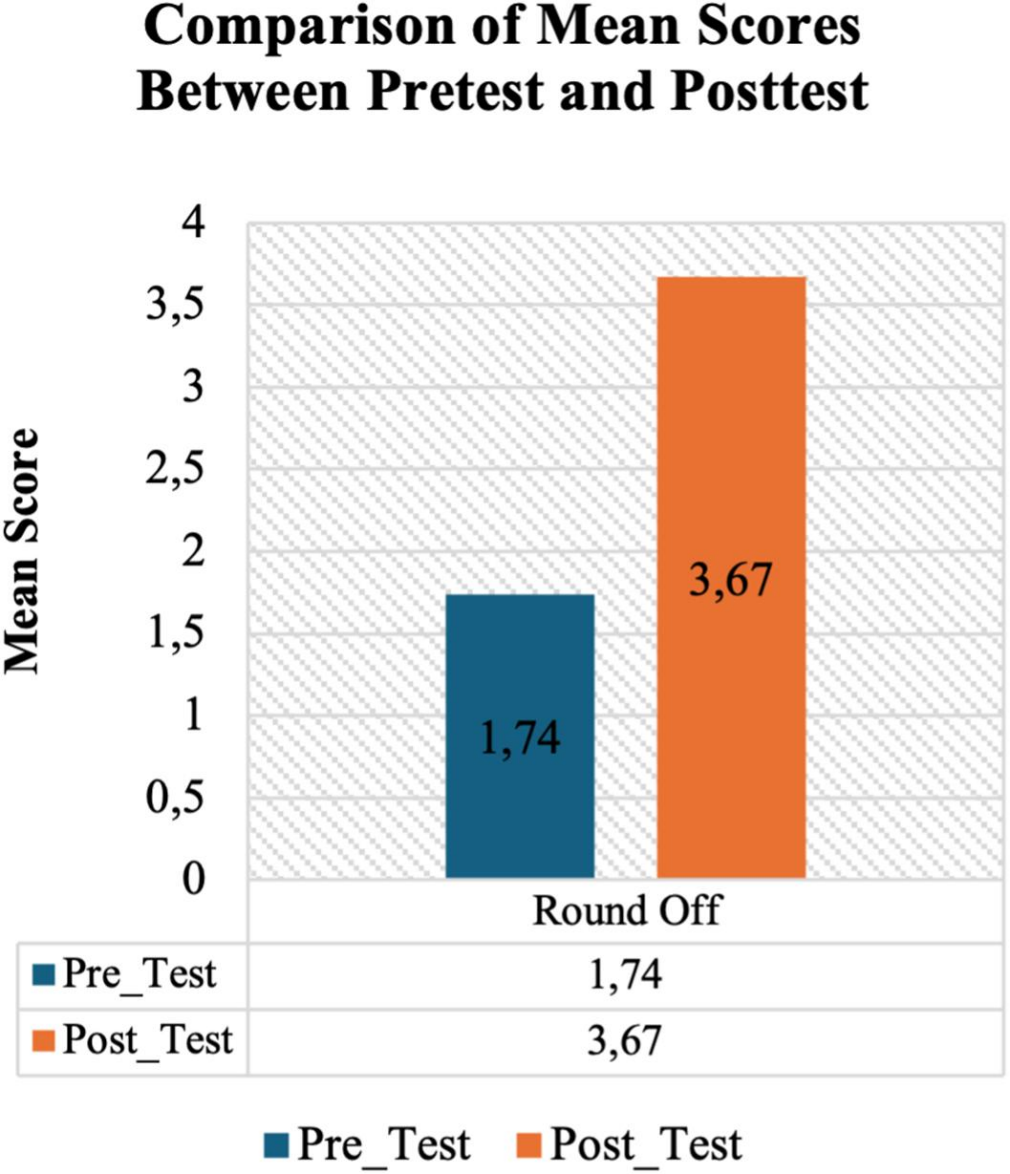
Comparison of mean scores between pretest and posttest of round-off learning outcomes.

Table 1 above shows us how much better students got at doing round-off movements before and after they used a smartphone video analysis app as part of their treatment. The study included 36 students as subjects. The pre-test results had a mean of 1.74 and a standard deviation of 0.96. This means that before treatment, students’ ability to perform round-off was in the low to moderate range, with a fairly wide range of values (from 1.00 to 3.50). After treatment, the average post-test score rose significantly to 3.67, with a lower standard deviation of 0.48. The lowest score went up to 2.00 and the highest score went up to 4.00. This means that almost all of the students showed high or almost maximum abilities after using the video analysis app. The lower standard deviation value in the post-test also shows that technology-assisted learning made students’ skills more consistent and similar. This shows that using video analysis apps on smartphones can really help students improve their round-off movement skills. Figure 1 shows the difference between the average scores on the pretest and posttest.

After determining the average scores for each subject, statistical calculations were performed to determine the effect of using the sport motion analysis smartphone app on roundoff learning outcomes. The results are shown in Table 2.

**Table 2:** Statistical calculation results.

Variabel	N	Z-value	*p*-value	Effect Size (r)	Interpretasi
Round-Off	36	−5.18	0.00	0.86	Large effect

*P*-value < 0.05, H0 Rejected, Accepted H1.

*P*-value > 0.05, H0 Accepted, Rejected H1.

The Wilcoxon test shows a Z value of −5.18, and the Sig (2-tailed) value is less than 0.05. This means that H1 is accepted and Ho is rejected. This indicates a substantial correlation between the pre-test and post-test. The table of Test Statistics results above shows that the Wilcoxon Signed-Rank Test was used to see if there was a difference between the two paired measurements, which were the students’ pre-test and post-test results for their round-off skills. Alongside statistical significance, the intervention impact's magnitude was assessed on the effect size. The effect size for the Wilcoxon signed-rank test was determined using the r statistic, resulting in a value of r = 0.86, which is classified as a large effect per Cohen's criteria. This finding demonstrates that the enhancement in round-off performance from pre-test to post-test was both statistically significant and practically large, indicating a considerable change after the learning intervention.

## 4 Discussion

In the realm of motor skill acquisition, a distinctive principle asserts that observation can facilitate the adoption of novel motor behaviors that exceed an individual's current capabilities (10). As the first step in a new movement learning activity, teachers must show students how to do it. Giving feedback is very important in this activity, especially for movements that are hard to do. The goal is to let students know if the movements they are doing are right or wrong. Teachers need to give students feedback in the form of videos, either live or recorded, so that they can understand the movements they are learning. Video is an important tool for teaching and learning because it uses both sides of the brain (25). Videos can also motivate students to make similar video projects (25). The students are making a video as part of a series of lessons on how to do round-off locomotor movements using a video analysis app on their phones. Using mobile video analysis tools in physical education has many benefits (26). One benefit for teachers is that it makes it easy to give students immediate feedback on how well they are doing (27). This can help students learn motor skills that fit their different needs (23).

Students make videos of themselves doing round-off exercises, and then they use an app to look at the videos and figure out what they did wrong. The analysis is done by comparing the movements that were made to the right round-off movements. This is done with this video analysis app by drawing lines on body parts that aren't moving the way they should. This is to find mistakes and figure out how to fix the movement. With this app, students can also see the angle of the movement they did and talk about what went wrong and how to fix it. The Round-off movement has three parts: (1) the running stage, where you raise your hands one at a time and put them on the floor while your body turns; (2) the main stage, where you turn your hands 1/4 of the way around the wheel; and (3) the final stage, where you put both feet together back on the floor at the same time, jump together, and then do another movement (21).

Students watch an expert do the round-off movement before they start the video analysis process. The goal is to look at the student's movement video and the expert's video side by side. Adding video modeling by an expert with video feedback during practice can cut down on the number of times you need to practice difficult physical skills (15). You can use a video analysis app to see how well someone is doing by looking at the accuracy and angles formed by each body part when they do the movement. You can do this by adding an angle line to the app so that the angles formed can be seen. Then, students can match their movements with those of experts by changing the angles formed by experts when they do the movement. A study found that students could imitate the movements of experts faster when they did them themselves and then imitated them (28–30).

Numerous studies validate that the efficacy of video feedback is attributed to its capacity to deliver objective performance data, thereby assisting both learners and coaches in making informed decisions about training strategies (31, 32). Using video feedback, students can find flaws in their technique and change how they practice, which helps them understand the skill better and master it. Additional empirical evidence indicates that video feedback is especially beneficial for novices acquiring intricate motor skills, such as those required in gymnastics (5, 21, 33). In practice, learning also involves peers through video feedback. Research indicates that peer feedback mechanisms facilitate reciprocal learning, enhance observational skills, and promote reflective practice (5, 6). This collaborative approach not only helps students remember what they learned, but it also helps them understand performance quality in a more critical way, so they can see what they do well and what they need to work on in their own techniques (34, 35).

Another interesting thing about video feedback is that it can be used in a variety of learning settings. Numerous studies underscore the significance of contextual factors, including the student's learning stage, in assessing the efficacy of video feedback. For instance, Ferracioli et al. (7) found that video feedback can have a big effect on how quickly someone learns a skill, depending on whether they are a beginner or have already learned some rhythmic gymnastics skills. These results underscore the necessity for a customized approach to feedback mechanisms that considers the context and individual requirements of each learner, thereby optimizing the efficacy of video feedback on motor skill development (36, 37).

Studies have demonstrated that multidimensional feedback modalities, such as 3D visualization in conjunction with conventional video, can markedly enhance the quality of gymnastics movement execution, thereby reinforcing the efficacy of video feedback. This indicates that various feedback modalities can effectively address the intricate dynamics of skill acquisition in gymnastics, facilitating a more thorough learning experience (21, 36). The integration of video modeling and real-time feedback frequently improves the learning process by enabling students to correlate their movements with those of experts, thereby establishing a more defined framework for skill acquisition (38, 39). Studies investigating performance feedback from experts and peers indicate that the capacity to critically observe and analyze performance facilitates students in identifying and rectifying their own errors efficiently (5, 40).

The authors recognize that this study utilized a single-group quasi-experimental pretest–posttest design lacking a control group. Although changes in learning outcomes were noted over time, this approach constrains the capacity to make robust causal conclusions about the intervention's success. The noted enhancements may have been affected by instructional advice, natural learning development, or the impacts of repeated practice, rather than only the intervention.

The authors recognize that this study utilized an observation checklist to evaluate round-off performance; nonetheless, the comprehensive psychometric features of the instrument were neither thoroughly assessed nor reported. Specifically, data about the instrument's validity, inter-rater reliability, and intra-rater reliability was not established, nor was formal evaluator training consistently recorded. As a result, statistical reliability metrics such as the intraclass correlation coefficient (ICC) or Cohen's kappa were not computed. This constraint may influence the consistency and impartiality of the evaluation outcomes, and subsequent research is urged to utilize validated instruments and disclose thorough reliability statistics.

## 5 Conclusion

This study's results indicated favorable outcomes in acquiring the round-off locomotor movement through a smartphone application. Students were able to understand the feedback provided by the teacher well during the lesson. Video technology has generally been shown to be an effective, equitable, and flexible method for teaching physical education, facilitating the improvement of students’ motor skills, particularly in precision-based activities such as the round-off.

The authors recognize that the study's sample size was limited, comprising only 36 students from one university, which may constrain statistical power and diminish the generalizability of the findings.

Moreover, the participants possessed previous gymnastics experience, complicating the generalization of the results to novice learners or different educational contexts. These factors may influence the overall external validity of the study. Moreover, the absence of a control group permits the emergence of unforeseen external confounding variables, rendering the results demonstrative rather than definitive. Future research should utilize larger, more diverse samples, incorporate a control group, and conduct *a priori* power analysis to increase statistical robustness and generalizability.

Further research is recommended to assess the effectiveness of video feedback across skill levels, including comparisons between beginner, intermediate, and advanced athletes, to examine differences in learning responses. Additionally, future research could examine the use of advanced technologies—such as AI-based motion analysis or 3D visualization—to determine whether these methods lead to improved learning outcomes. Research could also expand the skill context to other sports or investigate the long-term effects of video feedback on motor skill retention.

## Data Availability

The dataset includes video recordings of participants, which cannot be made publicly available due to confidentiality and privacy concerns. Access to anonymized data may be granted by the corresponding author upon reasonable request and with appropriate ethical approval. Requests to access the datasets should be directed to rizalafauzi13@upi.edu.
