# Correction: The effect of digital video feedback on roundoff learning outcomes in gymnastics learning

**DOI:** 10.3389/fspor.2026.1846652

**Published:** 2026-04-22

**Authors:** Rizal Ahmad Fauzi, Ayi Suherman, Entan Saptani, Suthana Tingsabhat, Mamat Heryanto

**Affiliations:** 1 Physical Education Study Program, Universitas Pendidikan Indonesia, Bandung, Indonesia; 2 Department of Curriculum and Instruction, Faculty of Education, Chulalongkorn University, Bangkok, Thailand; 3 Department of Physical Education, Health and Recreation STKIP Pasundan, Cimahi, Indonesia

**Keywords:** digital video, feedback, gymnastics learning, round-off, technology

The funding information was omitted in the original version of this article.

The correct funding statement is as follows:

“This research was funded by the Indonesian Endowment Fund for Education (LPDP), on behalf of the Ministry of Higher Education, Science and Technology of Indonesia, and managed under the EQUITY Program (Contract No. 4314/B3/DT.03.08/2025 and No. 352/UN40/HK.00.10/2025).”

The original version of this article has been updated.

